# Genetic architecture and biology of youth-onset type 2 diabetes

**DOI:** 10.1038/s42255-023-00970-0

**Published:** 2024-01-26

**Authors:** Soo Heon Kwak, Shylaja Srinivasan, Ling Chen, Jennifer Todd, Josep M. Mercader, Elizabeth T. Jensen, Jasmin Divers, Amy K. Mottl, Catherine Pihoker, Rachelle G. Gandica, Lori M. Laffel, Elvira Isganaitis, Morey W. Haymond, Lynne L. Levitsky, Toni I. Pollin, Jose C. Florez, Jason Flannick, Progress in Diabetes Genetics in Youth (ProDiGY) consortium

**Affiliations:** 1 Department of Internal Medicine, Seoul National University Hospital, Seoul National University College of Medicine, Seoul, Republic of Korea; 2 Programs in Metabolism and Medical and Population Genetics, Broad Institute of MIT and Harvard, Cambridge, MA USA; 3 Division of Pediatric Endocrinology, University of California at San Francisco, San Francisco, CA USA; 4 Center for Genomic Medicine, and Diabetes Unit, Endocrine Division, Department of Medicine, Massachusetts General Hospital, Boston, MA USA; 5 Division of Pediatric Endocrinology, Department of Pediatrics, University of Vermont, Burlington, VT USA; 6 Department of Medicine, Harvard Medical School, Boston, MA USA; 7 Department of Epidemiology and Prevention, Wake Forest School of Medicine, Winston-Salem, NC USA; 8 Department of Foundations of Medicine, NYU Langone Health, New York, NY USA; 9 Division of Nephrology and Hypertension, University of North Carolina, Chapel Hill, NC USA; 10 Department of Pediatrics, University of Washington, Seattle, WA USA; 11 Naomi Berrie Diabetes Center, Columbia University Irving Medical Center, New York, NY USA; 12 Joslin Diabetes Center, Harvard Medical School, Boston, MA USA; 13 Department of Pediatrics, Children’s Nutrition Research Center, Baylor College of Medicine, Houston, TX USA; 14 Pediatric Endocrine Division, Department of Pediatrics, Massachusetts General Hospital for Children and Harvard Medical School, Boston, MA USA; 15 Division of Endocrinology, Diabetes, and Nutrition, Department of Medicine, University of Maryland School of Medicine, Baltimore, MD USA; 16 Division of Genetics and Genomics, Boston Children’s Hospital, Boston, MA USA; 17 Department of Pediatrics, Boston Children’s Hospital, Boston, MA USA

**Keywords:** Diabetes, Population genetics, Metabolism, Genetics, Endocrine system and metabolic diseases

## Abstract

The prevalence of youth-onset type 2 diabetes (T2D) and childhood obesity has been rising steadily^1^, producing a growing public health concern^1^ that disproportionately affects minority groups^2^. The genetic basis of youth-onset T2D and its relationship to other forms of diabetes are unclear^3^. Here we report a detailed genetic characterization of youth-onset T2D by analysing exome sequences and common variant associations for 3,005 individuals with youth-onset T2D and 9,777 adult control participants matched for ancestry, including both males and females. We identify monogenic diabetes variants in 2.4% of individuals and three exome-wide significant (*P* < 2.6 × 10^−6^) gene-level associations (*HNF1A*, *MC4R*, *ATXN2L*). Furthermore, we report rare variant association enrichments within 25 gene sets related to obesity, monogenic diabetes and β-cell function. Many youth-onset T2D associations are shared with adult-onset T2D, but genetic risk factors of all frequencies—and rare variants in particular—are enriched within youth-onset T2D cases (5.0-fold increase in the rare variant and 3.4-fold increase in common variant genetic liability relative to adult-onset cases). The clinical presentation of participants with youth-onset T2D is influenced in part by the frequency of genetic risk factors within each individual. These findings portray youth-onset T2D as a heterogeneous disease situated on a spectrum between monogenic diabetes and adult-onset T2D.

## Abstract (web summary)

Kwak et al. identify a mixture of monogenic, rare and common genetic variants of youth-onset type 2 diabetes (T2D), highlighting the heterogeneity of youth-onset T2D and positioning it on a genetic spectrum between monogenic diabetes and adult-onset T2D.

## Main

Given its clinical presentation and early age of onset, youth-onset T2D could be proposed to be caused by (1) increased environmental risk on a genetic background similar to^4^ or at the extremes of^5^ adult-onset T2D, (2) reduced penetrance of monogenic diabetes variants^6^ or (3) risk factors not shared with adult-onset or monogenic diabetes. These risk factors may implicate similar genes and pathways to those for adult-onset and monogenic diabetes, but this is far from certain, particularly given that individuals with youth-onset T2D are often obese^1^ and therefore clinically distinct from those with early-onset monogenic diabetes caused by β-cell dysfunction^7^ or severe insulin resistance^8^. Two recent studies from the Progress in Diabetes Genetics in Youth (ProDiGY) Consortium provided glimpses into youth-onset T2D genetic risk, showing that affected individuals are both enriched for adult-onset T2D common variant polygenic risk^4^ and include undiagnosed monogenic diabetes^9^. However, the relative contribution of adult-onset and monogenic diabetes genetic risk factors to youth-onset T2D remains unknown, as does the extent of youth-onset T2D genetic and biological heterogeneity^10^.

**Fig. 1:**
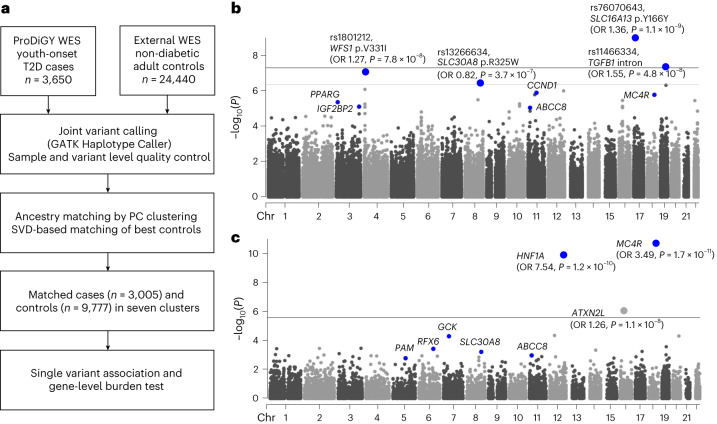
Scheme of the study and genetic discovery. **a**, Whole-exome sequence data of individuals with youth-onset T2D were matched to those of external non-diabetic control participants using genetic principal components and a singular-value decomposition (SVD)-based method resulting in 3,005 cases and 9,777 control participants for single-variant and gene-level association analysis. **b**, Single-variant association analysis revealed four variants passing exome-wide significance (*P* < 4.3 × 10^−7^). **c**, Gene-level association analysis showed three genes associated with youth-onset T2D at exome-wide significance (*P* < 2.6 × 10^−6^). Blue dots represent previously known variants or genes of adult-onset T2D. Both single-variant and gene-level association analyses were performed with Firth’s penalized logistic regression. GATK, genome analysis toolkit; PC, principal components; WES, whole-exome sequencing.

**Extended Data Fig. 1:**
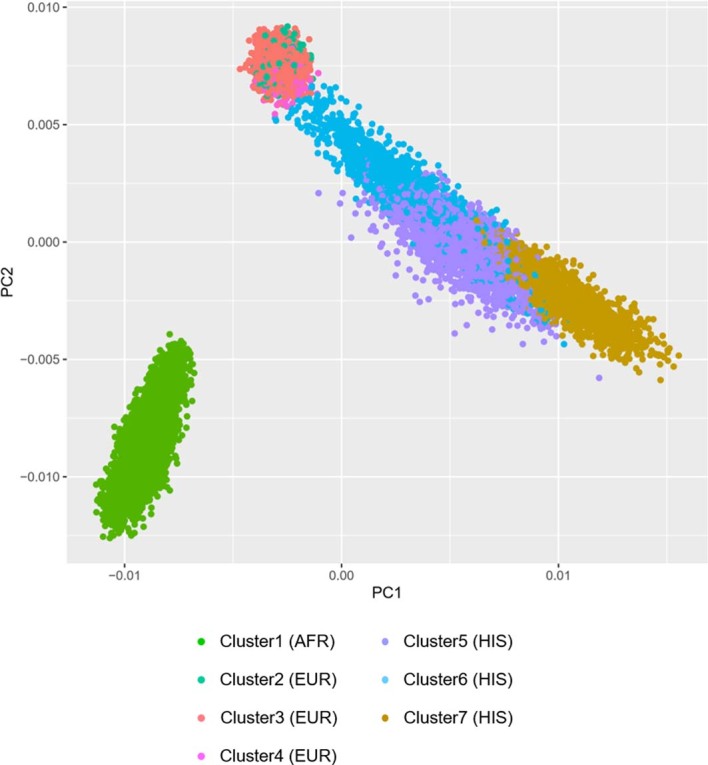
Principle components based matching of ancestry. Applying principal component analysis (PCs) and a singular-value decomposition-based method to match external controls with ProDiGY cases yielded seven clusters from three different ancestries. AFR, African; EUR, European; HIS, Hispanic.

**Extended Data Fig. 2:**
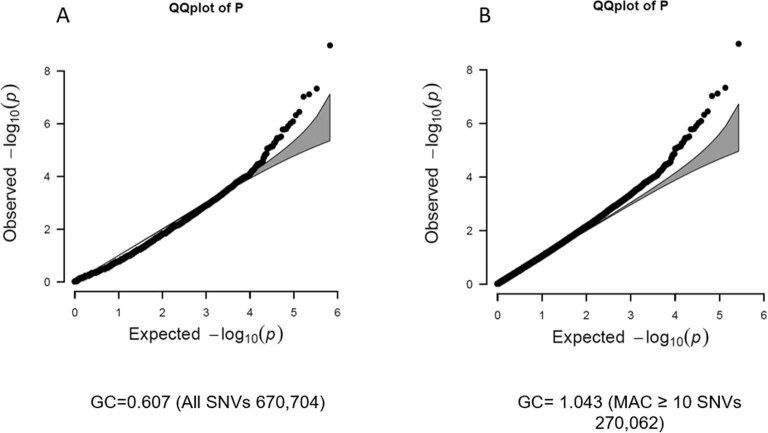
Quantile-quantile plot of the single variant association test. Quantile-quantile plot showing the distribution of the observed *P* values from the single variant association test against the expected distribution under the null hypothesis for all single nucleotide variants (A) and for variants with a minor allele count of 10 or more (B). The grey zone indicates the 95% confidence interval. GC, genomic inflation factor; MAC, minor allele count; SNV, single nucleotide variant.

**Extended Data Fig. 3:**
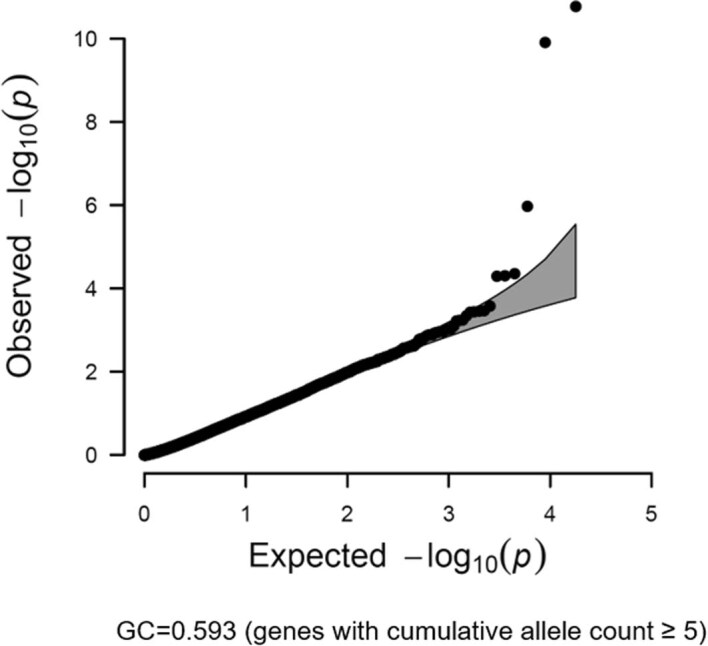
Quantile-quantile plot of the gene-level rare coding variant association test. Quantile-quantile plot showing the distribution of the observed *P* values from the gene-level rare coding variant association test against the expected distribution under the null hypothesis. The grey zone indicates the 95% confidence interval. GC, genomic inflation factor.

**Extended Data Fig. 4:**
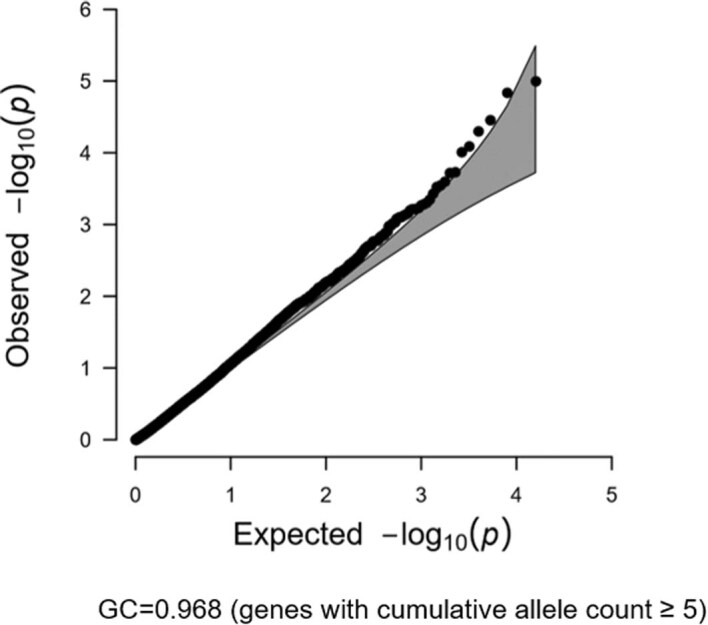
Quantile-quantile plot of the gene-level association test using synonymous variants. Quantile-quantile plot showing the distribution of the observed *P* values from the gene-level association test using synonymous variants against the expected distribution under the null hypothesis. The grey zone indicates the 95% confidence interval. GC, genomic inflation factor.

To further explore the genetic basis of youth-onset T2D and to localize the genetic risk to specific genes and pathways, we obtained youth-onset T2D exome sequence and genome-wide association study (GWAS) data from the ProDiGY Consortium (Fig. 1a and Methods). Focusing first on rare coding variants, we combined the exome sequences with non-diabetic adult control exome sequences from the recent AMP-T2D-GENES study^6^. Joint variant calling, quality control and genetic clustering and matching^11^ (Extended Data Fig. 1 and Methods) produced an analysis set of 3,005 individuals with youth-onset T2D and 9,777 control participants (African Americans *n* = 4,189, Europeans *n* = 2,546, Hispanics *n* = 6,047; Supplementary Table 1). We applied a previous methodology^12^ to conduct single-variant and gene-level association analyses (Methods), neither of which showed any evidence of systemic test statistic inflation (Extended Data Figs. 2 and 3) or rare synonymous variant associations (Extended Data Fig. 4).

**Extended Data Fig. 5:**
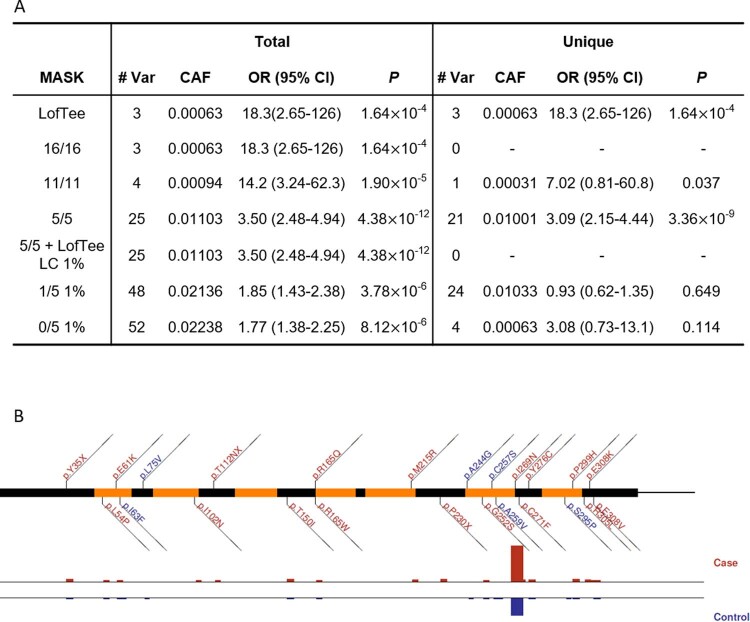
Gene-level analysis of *MC4R*. Shown is a dissection of the gene-level associations for *MC4R*. (A) Mask-level Firth’s logistic regression analysis results for all variants in the mask are shown in the left column (“Total”) and for variants unique to the mask are shown in the right column (“Unique”). The details of the mask definition is described in the Methods. (B) A graphical plot of variants observed in *MC4R* within the 1/5 1% mask. Variants are coloured blue (if individual OR < 1) or red (OR > 1). Case (red) and control (blue) frequencies are shown below for each variant. # Var, number of variants in the association test; CAF, combined allele frequency; OR, odds ratio.

**Extended Data Fig. 6:**
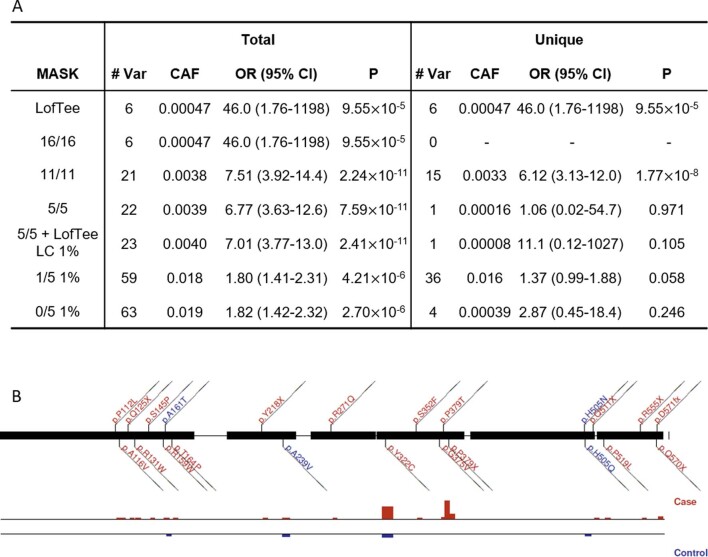
Gene-level analysis of *HNF1A*. Shown is a dissection of the gene-level associations for *HNF1A*. (A) Mask-level Firth’s logistic regression analysis results for all variants in the mask are shown in the left column (“Total”) and for variants unique to the mask are shown in the right column (“Unique”). The details of the mask definition is described in the Methods. (B) A graphical plot of variants observed in *HNF1A* within the 1/5 1% mask. Variants are coloured blue (if individual OR < 1) or red (OR > 1). Case (red) and control (blue) frequencies are shown below for each variant. # Var, number of variants in the association test; CAF, combined allele frequency; OR, odds ratio.

**Extended Data Fig. 7:**
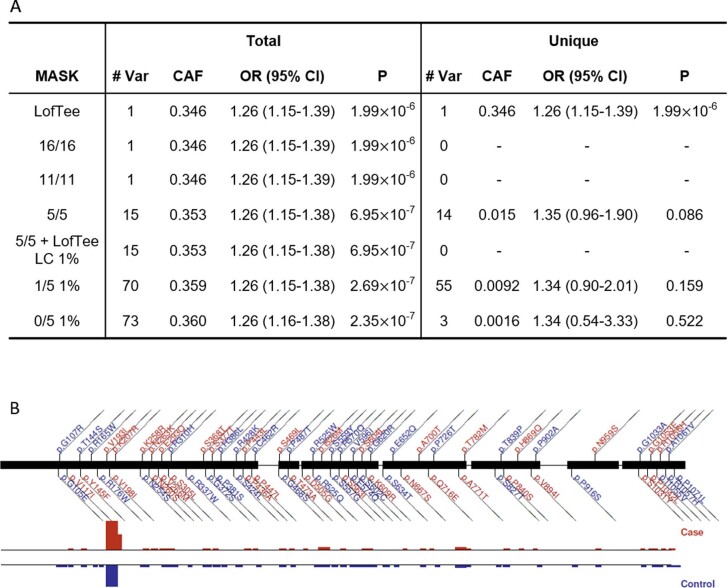
Gene-level analysis of *ATXN2L*. Shown is a dissection of the gene-level associations for *ATXN2L*. (A) Mask-level Firth’s logistic regression analysis results for all variants in the mask are shown in the left column (“Total”) and for variants unique to the mask are shown in the right column (“Unique”). The details of the mask definition is described in the Methods. (B) A graphical plot of variants observed in *ATXN2L* within the 1/5 1% mask. Variants are colored blue (if individual OR < 1) or red (OR > 1). Case (red) and control (blue) frequencies are shown below for each variant. # Var, number of variants in the association test; CAF, combined allele frequency; OR, odds ratio.

Four single variants (Fig. 1b and Supplementary Table 2) and three genes (*MC4R*, *HNF1A* and *ATXN2L;* Fig. 1c, Supplementary Tables 3 and 4 and Extended Data Figs. 5–7) showed exome-wide significant association^13^ (*P* < 4.3 × 10^−7^ for single-variant and *P* < 2.6 × 10^−6^ for gene-level associations; Methods). All four variants were common and previously associated with adult-onset T2D. For the gene-level associations, all but the *ATXN2L* association (odds ratio (OR) 1.26, 95% confidence interval 1.15–1.39, *P* = 1.1 × 10^−6^, combined minor allele frequency (MAF) 0.36 for 73 variants) have been previously reported as being associated with diabetes. The gene-level *ATXN2L* association was primarily due to a single common variant (rs55719896, *P* = 5.7 × 10^−5^) within an *ATXN2L* splice acceptor site and in strong linkage disequilibrium (*R*^2^ = 0.99) with an intronic variant (rs9972768) of *SH2B1* (a gene with a confirmed role in body mass index (BMI) variability), although none of the rare missense variants of *ATXN2L* were in linkage disequilibrium (*R*^2^ < 0.3) with variants in *SH2B1*. Further validation of this association is required to implicate *ATXN2L* in diabetes.

Notably, the six other significant associations had substantially larger effect sizes for youth-onset T2D than previously reported for adult-onset T2D. Compared with the previous AMP-T2D-GENES study for adult-onset T2D, the single-variant associations showed a mean OR increase of 1.14-fold (Supplementary Table 5), while the *MC4R* (OR 3.49, *P* = 1.7 × 10^−11^, combined MAF 0.011 for 25 variants) and *HNF1A* (OR 7.54, *P* = 1.2 × 10^−10^, combined MAF 0.0038 for 21 variants) gene-level associations had 1.69- and 6.13-fold higher effect sizes. Both the *MC4R* and *HNF1A* associations exhibited large effect sizes (OR ≥ 3) and achieved nominal significance in African American, European and Hispanic subgroups of youth-onset T2D (Supplementary Table 6). The large effect size was not solely attributed to the ProDiGY inclusion criteria (which required BMI ≥ 85th percentile for one cohort; Methods), since limiting the cases to a subset (*n* = 480) without this criterion also showed large effect sizes for both *MC4R* (OR 4.06) and *HNF1A* (OR 6.62).

To further explore the relationship between adult-onset and youth-onset T2D risk factors, we assembled broader collections of adult-onset T2D-associated coding variants and evaluated their associations in ProDiGY. First, among the 17 single variants with the strongest adult-onset T2D associations (*P* < 1.0 × 10^−5^) in the previous AMP-T2D-GENES (ref. ^6^) exome analysis, 13 showed nominal (*P* < 0.05) associations with youth-onset T2D in ProDiGY, all of which had consistent direction of effect between the two studies and larger effect sizes in ProDiGY compared to AMP-T2D-GENES (binomial *P* < 0.0001 accounting for control sample overlap, sample size difference and winner’s curse, 1.10-fold average increase; Methods and Supplementary Table 7). Second, among 38 genes in a ‘known diabetes gene set’ and with nominal (*P* < 0.05) gene-level associations in either AMP-T2D-GENES or ProDiGY (Methods and Supplementary Table 8), 27 had consistent directions of effect and 21 of these (77.8%) had larger effect sizes in ProDiGY (binomial *P* < 0.016 accounting for control sample overlap and sample size difference, 2.86-fold average increase; Supplementary Table 9). Thus, the strongest adult-onset T2D genetic risk factors (both common and rare variants) were also observed in youth-onset T2D, at a greater frequency in youth-onset T2D compared to adult-onset T2D.

To also explore the contribution of monogenic diabetes risk factors to youth-onset T2D, we curated six gene sets, designated as ‘monogenic OMIM + neonatal diabetes’, ‘monogenic primary’ (diabetes as a primary phenotype), ‘monogenic secondary’ (diabetes as a secondary phenotype), ‘monogenic obesity’, ‘lipodystrophy’ and ‘type 1 diabetes’ (Supplementary Table 10 and Methods). Wilcoxon rank-sum tests of youth-onset T2D associations showed that the ‘Monogenic OMIM + neonatal diabetes’ gene set with 19 genes for maturity-onset diabetes of the young (MODY) and neonatal diabetes showed the strongest gene-set enrichment (5.69-fold enrichment, *P* = 6.2 × 10^−5^). The set of 37 monogenic obesity genes showed a weaker but also nominally significant enrichment (2.05-fold enrichment, *P* = 0.034). The other four gene sets were not significantly associated with youth-onset T2D (*P* > 0.10), suggesting that youth-onset T2D is unlikely to primarily represent a collection of rare syndromic forms of diabetes. In addition to the lack of rare variant associations within the type 1 diabetes gene set, there were no significant youth-onset T2D common variant associations that aligned with a known direction of association for recognized type 1 diabetes (Supplementary Tables 11 and 12). These results indicate that youth-onset T2D shares genetic variants and genes overlapping those for some, but not all, forms of diabetes.

**Fig. 2:**
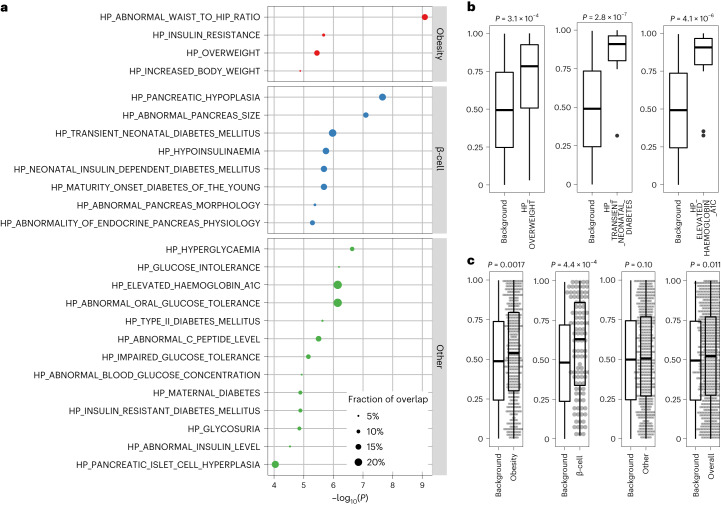
Pathways involved in obesity and β-cell function are enriched in youth-onset T2D. **a**, Gene-set enrichment analysis using a hypergeometric test with the top 50 gene-level association signals in youth-onset T2D identified 25 Human Phenotype Ontology gene sets that had significant overlap and were related to metabolic phenotypes of diabetes. These 25 gene sets were categorized into three subgroups of ‘obesity’, ‘β-cell function’ and ‘others’. **b**, A one-sided Wilcoxon rank-sum test (one-sided) using these 25 gene sets revealed representative sets with significant association enrichments beyond the top 50 associated genes, such as ‘HP_OVERWEIGHT’ (*n* = 24 genes versus 1,132 background genes), ‘HP_TRANSIENT_NEONATAL_DIABETES_MELLITUS’ (*n* = 16 genes versus 750 background genes), and ‘HP_ELEVATED_HAEMOGLOBIN_A1C’ (*n* = 15 genes versus 705 background genes). **c**, Gene-set clusters of ‘obesity’ (*n* = 438 genes versus 1,999 background genes) and ‘β-cell function’ (*n* = 108 genes versus 519 background genes) showed significant enrichment (*P* < 0.05) when combining genes across all sets in the cluster using the one-sided Wilcoxon rank-sum test. Background denotes matched genes with similar numbers and frequencies of variants within them. All box-and-whisker plots represent the following: line, median; box, interquartile range (IQR) and whiskers, 1.5 × IQR.

**Extended Data Fig. 8:**
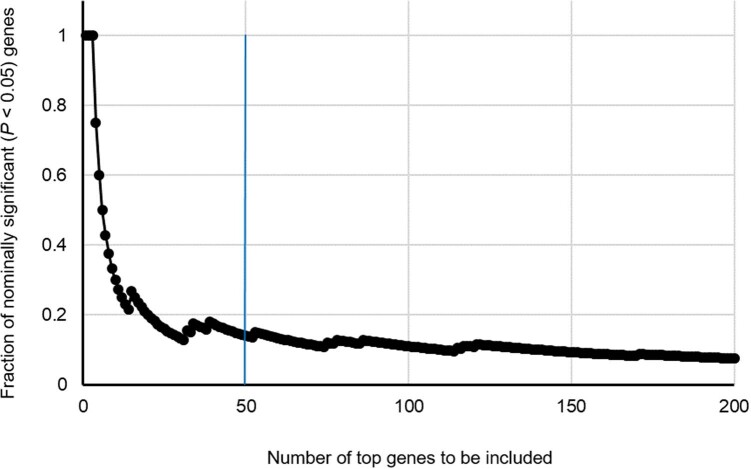
Number of top genes of youth-onset T2D and their fraction of nominally significant associations in AMP-T2D-GENES. To determine the cutoff for the number of top genes to be included in the gene set enrichment analysis, we examined the proportion of genes associated with T2D in the AMP-T2D-GENES database that reached nominal significance. The top 50 genes from our gene-level association study of youth-onset T2D demonstrated an enrichment of established T2D association signals.

To investigate biological mechanisms underlying youth-onset T2D, we conducted rare variant association enrichment tests across 5,071 gene sets delineated by Human Phenotype Ontology^14^ terms and curated from the Molecular Signature Database^15,16^ (Methods). We examined the 50 most significant youth-onset T2D gene-level associations, a threshold above which youth-onset T2D associations demonstrated increased replication in adult-onset T2D associations (AMP-T2D-GENES) (Supplementary Table 13, Extended Data Fig. 8). Thirty-eight gene sets showed significant enrichment (*q* value < 0.01; Supplementary Table 14), 25 of which were related to metabolic phenotypes (for example, ‘diabetes’, ‘hyperglycaemia’, ‘overweight’, ‘waist’, ‘insulin’, ‘c peptide’, Methods). Of these 25, 14 were significantly enriched for youth-onset T2D associations under the Wilcoxon rank-sum test (*P* < 0.002 for multiple comparison), and these were grouped into three clusters: ‘obesity’ (for example, ‘HP_OVERWEIGHT’), ‘β-cell function’ (for example, ‘HP_TRANSIENT_NEONATAL_DIABETES_MELLITUS’) and ‘other T2D’ (for example, ‘HP_ELEVATED_HAEMOGLOBIN_A1C’; Fig. 2a,b and Supplementary Table 15). The ‘obesity’ and ‘β-cell function’ clusters (but not the ‘other T2D’ cluster) in fact showed significant enrichments (*P* < 0.05) when we combined genes across all sets in the cluster (Fig. 2c). These associations were not solely driven by the top 50 youth-onset T2D genes, as 16 gene sets remained significant (*P* < 0.05) even after removing the top 50 genes (Supplementary Table 16).

As this provided evidence for rare variant youth-onset T2D risk factors to be spread across many genes but falling short of exome-wide significance in our current analysis, we used the ProDiGY rare variant gene-level and gene-set associations to categorize genes into four tiers, each potentially harbouring rare variant risk factors for youth-onset T2D (Methods and Supplementary Table 17). In particular, ‘tier 3’ included 11 genes among the top 50 youth-onset T2D associations and within at least two of the significantly enriched gene sets (Supplementary Table 18); ten of these genes (all but *ATXN2L*) had significant rare variant associations. The gene-set associations and prioritized genes illustrate youth-onset T2D to be biologically similar to monogenic diabetes and adult-onset T2D, with some notable contrasts. First, the clustering of enriched gene sets into three subgroups of ‘obesity’, ‘β-cell function’ and ‘other T2D’ suggests that youth-onset T2D is more biologically heterogeneous than other early-onset forms of diabetes such as monogenic diabetes or lipodystrophies. Second, three ‘tier 3’ genes (*SIX3*, *HESX1*, *GHRL*) had strongly suggestive evidence of association in ProDiGY despite showing no evidence of association for adult-onset T2D (all had *P* > 0.69 in AMP-T2D-GENES) and no previous links to monogenic diabetes. Finally, the protective ProDiGY association in *SLC30A8* (OR 0.35, *P* = 6.0 × 10^−4^) had a larger effect size than observed in AMP-T2D-GENES, suggesting that youth-onset T2D risk is influenced not only by risk-increasing variants but also by a depletion (even relative to adult-onset cases) of protective variants.

We next sought to compare the population-level relative contributions of monogenic, rare and common variants to youth-onset T2D. We first integrated our rare variant associations with curated lists of pathogenic or likely pathogenic monogenic diabetes variants according to the latest guidelines provided by the ClinGen Variant Curation Monogenic Diabetes Expert Panel (https://cspec.genome.network/cspec/ui/svi/affiliation/50016), with additional calibration of the results of multiple computational predictions^17^. Extending results reported previously^9^, we identified 72 (2.4%) youth-onset T2D individuals to have pathogenic and likely pathogenic variants of monogenic diabetes. Additionally, four of the ten monogenic diabetes genes (*HNF1A*, *GCK*, *ABCC8* and *INS*) exhibited *P* < 0.05 rare variant associations with youth-onset T2D in our primary gene-level analysis (which included both pathogenic and likely pathogenic and additional variants; Supplementary Table 19). Two of these genes (*HNF1A* and *GCK*) remained significant (*P* < 0.05) even after pathogenic and likely pathogenic variants were removed from analysis (Supplementary Tables 20–22). This suggests that youth-onset T2D is caused not only by (presumably undiagnosed) monogenic diabetes but also an expansion of the allelic series beyond what has been previously observed for monogenic diabetes^18^ and adult-onset T2D (ref. ^19^).

**Fig. 3:**
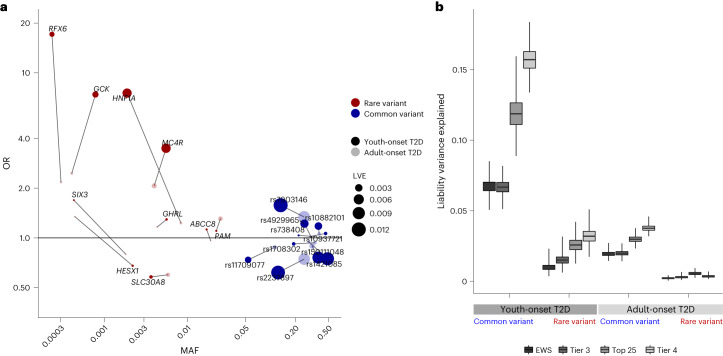
Genetic architecture and LVE by common and rare variants. **a**, OR, allele frequency distribution and LVE by ten tier 3 gene-level association signals and ten common variant association signals and their LVE in youth-onset T2D and adult-onset T2D. **b**, LVE by common variants and gene-level associations in youth-onset T2D and adult-onset T2D for exome-wide significant associations (EWS), ten tier 3 genes and same number of common variants (tier 3), top 25 significant gene-level and common variant associations (top 25) and 46 tier 4 genes and same number of common variants (tier 4). The LVE by common variants increased by 3.5–4.2-fold in youth-onset T2D compared to adult-onset T2D. There was even larger 5.0–9.0-fold increase in LVE by rare variant gene-level associations in youth-onset T2D. Box-and-whisker plots represent the following: line, median; box, IQR; whiskers, minimum and maximum.

To next compare liability variance explained (LVE) by the strongest youth-onset T2D rare coding variant and genome-wide common variant associations, we integrated our exome sequence data with common variant associations from the previous ProDiGY GWAS^4^. After correcting for winner’s curse and other confounders (Methods), the ten strongest ProDiGY GWAS common variant associations collectively had 4.5-fold larger LVE (6.7%) than did the ten ‘tier 3’ gene-level rare variant associations (1.5%) (Fig. 3a and Supplementary Table 23), indicating that (similar to adult-onset T2D) common variants are the dominant genetic risk factor for youth-onset T2D. However, compared to an equivalent analysis for adult-onset T2D in AMP-T2D-GENES (using comparable gene and variant sets and correcting for winner’s curse, different sample sizes and control sample overlap; Methods), youth-onset common variant LVE was 3.4-fold larger (*P* < 0.0001) than for adult-onset T2D (2.0%, Supplementary Tables 23 and 24), and youth-onset rare variant LVE was 5.0-fold larger (*P* < 0.0001) than for adult-onset T2D (0.3%). We repeated these analyses across three alternative sets of gene-level and GWAS associations and with adjustment for multiple potential confounders (Methods), observing the same trends: specifically, that youth-onset T2D associations, relative to adult-onset T2D associations, showed (1) larger absolute LVE by both common variants and rare variants and (2) a greater fold increase in rare variant LVE (5.0–9.0-fold increase) compared to common variant LVE (3.5–4.2-fold increase; Methods, Supplementary Tables 25–27 and Fig. 3b). These results support a model in which youth-onset T2D is enriched for genetic risk factors of all frequencies—with stronger enrichments for rarer variants—compared to adult-onset T2D.

**Fig. 4:**
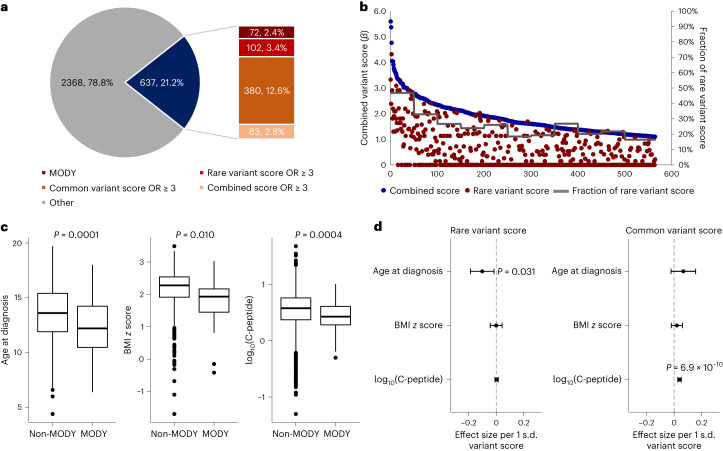
Individual genetic risk conferred by common and rare variants. **a**, Fraction of individuals having high genetic risk conferred by MODY variants, rare variant score, common variant score or combined variant score. Among 3,005 youth-onset T2D cases, 2.4% carried MODY variants, 3.4% had high rare variant score with OR ≥ 3, 12.6% had high common variant score with OR ≥ 3 and 2.8% had high combined score with OR ≥ 3. **b**, For the 565 non-MODY individuals having a high combined variant score with OR ≥ 3, the contribution of rare variant score was higher at the higher end of the combined variant score. **c**, Individuals with monogenic diabetes (*n* = 72) had an earlier age of diagnosis, lower BMI *z* score and lower log_10_(C-peptide) level compared to cases without MODY variants (*n* = 2,933). The difference in means between the two groups was tested using a generalized linear model. Box-and-whisker plots represent the following: line, median; box, IQR; whiskers, 1.5 × IQR. **d**, In linear regression analysis, rare variant score was associated with earlier age at diagnosis and common variant score was associated with higher log_10_(C-peptide) level even after excluding MODY cases (*n* = 2,933). Error bars indicate 95% confidence interval.

To investigate the genetic heterogeneity of individual-level youth-onset T2D, we computed sample-level ‘contribution scores’ (Methods) from monogenic variants, rare coding variants, genome-wide common variants or rare and common variants combined. Among the 3,005 ProDiGY participants, 637 (21.2%) had either a MODY variant or a combined score with OR ≥ 3 (Fig. 4a). These were a mixture of individuals with monogenic diabetes (72, 2.4%), rare variant OR ≥ 3 (102, 3.4%), common variant OR ≥ 3 (380, 12.6%) and combined OR ≥ 3 (83, 2.8%), suggesting individual-level genetic heterogeneity in terms of which risk factors predominate. Among the 565 non-MODY participants, rare variant scores were on average 25.1% of the combined scores, declining steadily from 46.8% for the first 50 individuals with the strongest combined variant scores to 16.4% for the last 65 individuals with the weakest combined variant scores (Fig. 4b). There was no obvious boundary to classify individuals as having ‘high’ versus ‘low’ rare variant scores.

Comparing clinical presentations of the youth-onset T2D participants, we found those with MODY variants to have an earlier age of diagnosis (12.5 ± 2.4 versus 13.6 ± 2.3, *P* = 0.0001), lower BMI *z* score (1.77 ± 0.80 versus 2.18 ± 0.57, *P* = 0.010) and lower log_10_(C-peptide) level (0.43 ± 0.25 versus 0.55 ± 0.33, *P* = 0.0004) than those without MODY variants (Fig. 4c). Rare variant score (after removing participants with MODY) was associated with earlier age at T2D diagnosis (*β* −0.094 years per 1 s.d. increase in risk score, *P* = 0.031), but not with BMI *z* score or C-peptide level. (Fig. 4d). By contrast, the common variant score was associated with higher log_10_(C-peptide) level (*β* = 0.040 per 1 s.d. increase, *P* = 6.9 × 10^−10^). These findings indicate that clinical heterogeneity of youth-onset T2D is in part influenced by the frequency of contributing genetic risk factors.

In summary, we identified a variety of youth-onset T2D genetic risk factors with effect sizes much larger than any observed for adult-onset T2D. Our study has limitations. First, our sample size was large for a rare disease^20^ but modest for a common disease, requiring some of our analyses to include suggestive associations or the strongest, rather than all, genetic risk factors. Second, despite extensive validations of our external control matching strategy (Methods), some population stratification probably persisted between cases and control participants, particularly for rare variants^21^. Third, our comparisons between youth-onset and adult-onset T2D risk factors relied on the larger AMP-T2D-GENES study that shared control participants with our study, requiring extensive simulations and analytical calculations to control for sample overlap, winner’s curse in each study, and difference in sample sizes (Methods). Our conclusions, although consistent across numerous sensitivity analyses (Methods), are most robust when stated qualitatively.

We conclude that clinically diagnosed youth-onset T2D is influenced by—in order of importance—common variants, rare variants, and monogenic variants. Youth-onset T2D is consistent with models of an ‘extreme’ presentation of T2D (ref. ^22^) enriched for risk factors of all frequencies: to an extent that study of roughly 3,000 youth-onset participants produced stronger associations than that of roughly 20,000 adult-onset participants^6^. Rare variant risk factors show the strongest relative enrichment, but in absolute terms common variant risk factors still explain the most disease heritability. At the population level, youth-onset T2D therefore appears to share genetic features of both a common and rare disease.

These risk factors overlap with those for both adult-onset T2D and monogenic diabetes but notably not all forms of diabetes, including lipodystrophies or type 1 diabetes. They lie within multiple biological pathways, contributing to (at minimum) β-cell development, insulin secretion and obesity-related insulin resistance, indicating that obesity and impaired β-cell function both are large pathophysiological factors in youth-onset T2D. Within these pathways, we prioritized 11 genes as probably involved in youth-onset T2D, including three with no previous links to adult-onset T2D or monogenic diabetes. One of these 11 genes, *SLC30A8*, had a larger protective effect than was observed for adult-onset T2D, contrasting with the usual model in which early-onset disease cases are due mostly to high-effect risk-increasing variants.

Most intriguingly, the phenotype of youth-onset diabetes (age of onset, BMI and C-peptide level) seems to differ depending on whether genetic risk is due primarily to monogenic variants, common variants or rare variants. These results indicate that clinical heterogeneity may be due not only to the pathways in which genetic risk factors lie (that is, insulin secretion versus insulin resistance) but also to the frequency spectrum of genetic risk factors within an individual: a result potentially consistent with models of common disease caused by both ‘core’ and ‘peripheral’ genes^23^. There are no clear dividing lines to classify individuals into different subtypes on the basis of risk variant frequency, and the clinical relevance of causal variant frequency is probably far less than that of patient clustering on the basis of observed phenotypes^10^ or genetic disease mechanism^24^, but this genetic model for youth-onset T2D may help to better understand and categorize not only youth-onset but adult-onset and monogenic diabetes as well. More broadly, it is likely that other diseases may have ‘intermediate’ phenotypes^25^ whose analysis—by combining the strengths of rare and common disease analyses—may help illuminate the probably blurred line between phenotypically related disease forms.

## Methods

### Study participants

ProDiGY is a collaborative effort to understand the genetic predisposition of youth-onset T2D using multi-ethnic diabetes cases from SEARCH, TODAY and the TODAY Genetics study as previously described^4,9^. In brief, SEARCH is a longitudinal observation study on youth-onset diabetes in the United States (diagnosed at under 20 years of age) initiated in 2000 (ref. ^26^). The TODAY study is a randomized clinical trial that enroled participants with T2D age 10–17 years between 2004 and 2009 (ref. ^27^). Participants were diagnosed with T2D before 18 years of age; had BMI ≥ 85th percentile for age, sex and height; and did not have evidence of type 1 diabetes (negative of pancreatic islet autoantibodies and positive for C-peptide level greater than 0.6 ng ml^−1^). The TODAY Genetics study is ancillary to the TODAY clinical trial and enroled additional cases with similar criteria to the TODAY study.

In the current study, we investigated a total of 3,650 individuals with youth-onset T2D (553 participants from SEARCH, 526 from TODAY and 2,571 from the TODAY Genetics study). Participants with confirmed MODY and those suspected to have MODY on the basis of clinical judgement (that is, autoantibody negative and not overweight or obese) at study enrolment were excluded. Non-diabetic adult control samples were derived from the previously published^6^ AMP-T2D-GENES whole-exome sequence analysis, which involved 29,791 T2D cases and 24,440 control participants from five major ancestries. Criteria for inclusion as non-diabetic control participants were study-specific and were described previously^6^. After matching cases and control participants on the basis of their genetic background as described below, there were 3,005 participants in total with youth-onset T2D and 9,777 control participants available for genetic association testing. The effective sample size of this study, defined as 4 × *n*_cases_ × *n*_controls_/(*n*_cases_ + *n*_controls_), was 9,194. In the case group, BMI was available in 881 participants whereas fasting C-peptide and age at diagnosis was available for 2,960 and 3,005 participants, respectively.

### Whole-exome sequence data generation

ProDiGY whole-exome sequencing data were generated as part of a previously published study and therefore used identical variant calling, quality control procedures and variant annotation procedures as described previously^6^. In brief, genomic DNA was extracted form peripheral leucocytes and was sheared, ligated with Illumina barcoded sequence adaptors and amplified. Whole-exome in-solution hybrid capture was done with the Illumina Rapid Capture Exome enrichment kit (target region size 38 Mb). The enriched libraries were quantified, normalized and subjected to massive parallel sequencing using the HiSeq 4000 Sequencing system. Sequencing reads were aligned to the human genome build hg19 using the Picard (https://broadinstitute.github.io/picard/), Burrows–Wheeler Alignment^28^ and Genome Analysis Toolkit^29^ software. We excluded duplicate or sex-discordant samples on the basis of identity-by-descent analysis, as well as lower call rate samples in any pair with an identity-by-descent value greater than 0.3.

### Matching external control participants

For matching ProDiGY samples to external control participants, we first used genetic principal components of 5,496 linkage disequilibrium pruned autosomal variants to cluster all case and control samples into ancestry groups. Clustering was performed using MClust Gaussian model fitting as implemented in the SVDFunctions package (https://github.com/alexloboda/SVDFunctions). We then applied a singular-value decomposition (SVD)-based method^11^ to find the best set of control participant-matching cases within each ancestry group. Specifically, left singular vectors of the case genotype matrix were used to compute the estimated residual norm for every prospective control and generate a ranking of the degree to which they represented cases within their ancestry group^11^. For every control residual vector norm threshold, an association test (between cases and controls above the threshold) was performed and a genomic inflation factor was estimated^11^. The largest control set that provided a genomic inflation factor less than 2.0 and control sample size of more than 500 were chosen for each ancestry cluster using the selectControls() function in the SVDFunctions package. There was a total of seven clusters (Extended Data Fig. 1) that had genomic inflation factors between 1.03 and 1.75, and the final genomic inflation factor after meta-analysis was 1.15.

### Variant annotation

We annotated variants using the ENSEMBL VEP (v.87)^30^. Annotations were generated for all ENSEMBL transcripts, using the –flag-pick allele option to designate a ‘best guess’ annotation to each variant on the basis of a set of ordered criteria for transcripts^31^: transcript support level (that is, supported by mRNA), biotype (that is, protein_coding), APPRIS isoform annotation (that is, principal), the deleteriousness of annotation (that is, preference given to transcripts with higher impact annotations), consensus coding sequence database status of the transcript^32^ (that is, a high-quality transcript set) and canonical status of the transcript and transcript length (that is, longer is preferred). We used the VEP LofTee (https://github.com/konradjk/loftee) and dbNSFP (v.3.2)^33^ plugins to yield additional bioinformatic predictions of variant deleteriousness. From the dbNSFP plugin, we extracted annotations from 15 distinct bioinformatic algorithms along with the mCAP algorithm^34^. Since these annotations were not transcript specific, we allocated them to all transcripts for the sake of downstream analysis. All single-variant analyses described in the paper use the ‘best guess’ annotation for each variant.

### Statistical analysis and reproducibility

To investigate genetic risk factors for youth-onset T2D, we conducted a series of analyses. These included single-variant and gene-level rare variant association studies, as detailed below. Additionally, we carried out gene-set enrichment analysis, examined LVE by these variants and analysed the individual-level genetic risk on the basis of common and rare variants also as described below.

### Single-variant association analysis

We performed single-variant association tests using Firth’s penalized logistic regression as implemented in Hail v.0.2.43 (https://github.com/hail-is/hail/releases/tag/0.2.43). Association testing was done for cases and matched control participants in each of the seven ancestry clusters, adjusting for genetic principal components (PC1–PC10) significantly associated with youth-onset T2D after Bonferroni correction (*P* < 0.005). For each cluster, we performed additional variant quality control by including only biallelic autosomal variants with (1) *P* ≥ 0.0001 for variant differential missingness between cases and control participants, (2) *P* ≥ 0.0001 for Hardy–Weinberg equilibrium, (3) (alternate allele genotype quality score (GQ) ≥ 95, call rate (CR) ≥ 0.95) or (alternate allele GQ < 95, CR ≥ 0.99, *P* ≥ 0.001 for variant differential missingness between cases and control participants, *P* ≥ 0.001 for Hardy–Weinberg equilibrium), (4) variant read depth greater than or equal to 50, or (variant read depth less than 50, *P* ≥ 0.001 for variant differential missingness between cases and control participants, *P* ≥ 0.001 for Hardy–Weinberg equilibrium, *P* ≥ 0.001 for Hardy–Weinberg equilibrium in cases, *P* ≥ 0.001 for Hardy–Weinberg equilibrium in controls), (5) Firth *P* ≥ 0.05 × *P* value for Fisher’s exact test and (6) a passing random forest filter of gnomAD exomes and genomes. We confirmed that these filters resulted in a well-calibrated test statistic for each cluster without significant inflation through inspection of quantile–quantile plots (Extended Data Fig. 2). Among the variants remaining within each cluster, we then conducted a seven-way inverse-variance weighted meta-analysis using METAL^35^ (some variants were present in fewer than seven clusters due to quality control exclusions). Exome-wide significance for coding variants^13^ was set as *P* < 4.3 × 10^−7^ and genome-wide significance for non-coding variants was set as *P* < 5.0 × 10^−8^. For downstream tests of concordance between youth-onset T2D effect sizes in ProDiGY and adult-onset T2D effect sizes in AMP-T2D-GENES, a binomial test was performed.

### Gene-level association analysis

Gene-level association analysis was conducted as previously described with minor modifications^6^. For each gene, we grouped variants into seven nested ‘masks’^6,36^ on the basis of 16 different bioinformatic predictions of variant deleteriousness^33^. These seven masks were (from most stringent to least stringent): (1) LOFTEE (LOFTEE high confidence), (2) 16 out of 16 (pass 11 out of 11 criteria, VEST3 > 90%, CADD > 90%, DANN > 90%, Eigen-raw > 90% and Eigen-principal component-raw > 90%), (3) 11 out of 11 (pass 5 out of 5 but fail 16 out of 16 criteria, FATHMM pred = D, FATHMM-MKL pre = D, PROVEAN pred = D, metaSVM pred = D, MetaLR = D and MCAP > 0.025), (4) 5 out of 5 (fail 11 out of 11 criteria, PolyPhen HDIV pred = D, PolyPhen HVAR pred = D, SIFT pred = del, LFT pred = D and MutTaster pred = D/A), (5) 5 out of 5 + LOFTEE LC 1% (pass 5 out of 5 criteria or VEP Impact = HIGH, LOFTEE low confidence and Max MAF < 1%), (6) 1 out of 5 1% (fail 5 out of 5 criteria, VEP Impact = MOD and Max MAF < 1%) and (7) 0 out of 5 1% (fail 1 out of 5 criteria, VEP Impact = MOD and Max MAF < 1%). For each gene and mask, up to three groupings of alleles were generated on the basis of different transcript sets of the gene. The variants included in each unique mask for the top 20 gene-level associations’ best guess transcript are displayed in Supplementary Table 4.

Before running gene-level association tests, we applied the same variant quality control filters as for single-variant association analysis. For each mask, we then conducted burden analyses (in which an individual’s phenotype is regressed on the number of variants in the mask carried by the individual) using Firth’s penalized logistic regression, as implemented in EPACTS v.3.2.4 (https://genome.sph.umich.edu/wiki/EPACTS). Regressions were adjusted for ten principal components and seven ancestry clusters. The seven *P* values for each gene mask and up to three *P* values for transcript sets were consolidated by a minimum *P* value test, in which a gene was assigned its smallest *P* value across masks after correction for the effective number of independent masks (as estimated by the gene-specific correlation of variants across masks^6,12^). As this procedure produced one *P* value per gene, the gene-level significance threshold was set to *P* < 2.6 × 10^−6^ (*P* = 0.05/19,020 genes).

### Accounting for sample size differences, control sample overlap and winner’s curse in effect size comparisons

Many of our analyses compared properties of associations across different datasets (ProDiGY versus AMP-T2D-GENES) and across frequency ranges (rare versus common). These properties included the proportion of associations in one study observed in the other, the consistency of effect size directions, the relative effect size magnitudes and the LVE. We applied a series of analytical and simulation-based adjustments for the potential confounders as below.

For comparisons of variant effect sizes between ProDiGY and AMP-T2D-GENES, we adjusted for control sample overlap and winner’s curse. We first simulated 1,000 replicates of two association studies (under the null model): one with the same number of cases and control participants as ProDiGY, one with the same number of cases and control participants as AMP-T2D-GENES and with the studies sharing the same number of control participants as the empirical studies. The number of variants and their frequencies were matched to the number empirically observed for ProDiGY. Genotypes were simulated as binomial random variables, cases had phenotypes set to 0 and controls had phenotypes set to one, and effect sizes and *P* values were generated via linear regression. We used the results of these studies to calculate expected values (and standard errors) for two quantities of interest in our analyses: effect size concordances between the studies, and fraction of variants that had larger effect sizes in one study as opposed to the other.

For analyses in which variants were ascertained on the basis of *P* values in either AMP-T2D-GENES or ProDiGY, we corrected for winner’s curse in these calculations by re-conducting these simulations for sets of variants chosen on the basis of either (1) their rank in the *P* value distribution for one of the two studies or (2) a *P* value threshold. This analysis accounted for differences in sample size (and hence winner’s curse) between the studies, depending on whether we drew simulated variants on the basis of their *P* values in the larger or smaller simulated study.

For analyses in which variants were not ascertained on the basis of their association results, and therefore not subject to winner’s curse, we simply drew a set at random from the simulations. For example, the ‘known diabetes gene set‘ was curated on the basis of various sources including genes causing monogenic diabetes, genes for T2D drug targets, genes identified in GWAS to harbour causal coding variants, genes of monogenic obesity and mouse diabetes genes as described in Supplementary Table 8.

### Accounting for winner’s curse for other analyses

For all other analyses in which we analysed variants selected on the basis of their observed *P* values in ProDiGY or AMP-T2D-GENES, we applied a previously developed winner’s curse correction to reduce the bias in effect size estimates^37^. Specifically, the likelihood of the observed effect size, conditional on achieving a given association threshold, is given by:

$$P\left({\beta }_{\mathrm{obs}}|{\beta }_{\mathrm{true}}\right)=\frac{\frac{1}{s}\phi \left(\frac{{{\beta }_{\mathrm{obs}}-\beta }_{\mathrm{true}}}{s}\right)}{\Phi \left(\frac{{\beta }_{\mathrm{true}}}{s}-c\right)+\Phi \left(\frac{{-\beta }_{\mathrm{true}}}{s}-c\right)}1\left(\left|\frac{{\beta }_{\mathrm{obs}}}{s}\right|\ge c\right)$$

where ${\beta }_{\mathrm{obs}}$ is the observed effect size, ${\beta }_{\mathrm{true}}$ is the winner’s curse corrected effect size (which we used in our downstream analyses), $c$ is the *z* score threshold (corresponding to the *P* value threshold) used to ascertain variants for the analysis, *s* is the standard error of ${\beta }_{\mathrm{obs}}$, $\phi$ is the normal density distribution and $\Phi$ is the normal cumulative distribution. We obtained ${\beta }_{\mathrm{true}}$ estimates using maximum likelihood (as implemented by numerical optimization). We obtained confidence interval estimates, under the asymptotic chi-squared approximation for the log-likelihood, by solving for the log-likelihood values that yielded the appropriate chi-squared distribution quantiles.

When calculating properties of a set of variants subject to winner’s curse (for example, total LVE explained), we obtained confidence intervals by sampling from the distributions of ${\beta }_{\mathrm{obs}}$. Specifically, for each sample, we drew a value of $\widetilde{{\beta }_{\mathrm{obs}}}$ for each variant by sampling from a chi-square distribution and then numerically finding the value of $\widetilde{{\beta }_{\mathrm{obs}}}$ with likelihood equal to the sampled value. We then calculated the property of interest (for example, LVE) using the sampled $\widetilde{{\beta }_{\mathrm{obs}}}$ values. We repeated this process 1,000 times and used the results to obtain desired confidence intervals.

### Gene-set enrichment analysis and gene-set classification

We conducted two types of enrichment analysis: one that evaluated the overlap of the set with the strongest gene-level associations, and the other that evaluated the entire set of gene-level *P* values in the set. For the first analysis, we analysed 5,071 Human Phenotype Ontology^14^ database gene sets as specified in MSigDB^16^. We used a hypergeometric test, as implemented in gene-set enrichment analysis (https://www.gsea-msigdb.org/gsea/index.jsp)^38^, to evaluate the overlap of each gene set with the top 50 genes from our ProDiGY analysis (that is, the 50 genes with the lowest *P* values according to the minimum *P* value test). We considered a gene set as significant if it achieved false discovery rate *q* value less than 0.01 on the basis of the Benjamini and Hochberg method.

Out of the 38 gene sets that showed significant over-representation of the top 50 gene-level association signals, 25 gene sets were defined by Human Phenotype Ontology terms including ‘diabetes’, ‘hyperglycaemia’, ‘glucose’, ‘HbA1c’, ‘insulin’, ‘pancreas’, ‘c peptide’, ‘overweight’ and ‘waist’. We considered these to be involved in metabolic phenotypes of T2D and further subdivided them into clusters of ‘β-cell function’, ‘obesity’ and ‘other T2D’ by independent analysis of two investigators. In cases of any conflicts, consensus was reached through discussion and agreement.

We subjected these 25 gene sets, as well as six others (‘Monogenic OMIM + neonatal diabetes’, ‘Monogenic primary’, ‘Monogenic secondary’, ‘Monogenic obesity’, ‘Lipodystrophy’ and ‘Type 1 diabetes’) described in Supplementary Table 10, to a second gene-set analysis. For each set we selected 50-fold matched control genes with similar numbers and frequencies of variants within them (following a previously described procedure^6^). We then performed a one-sided Wilcoxon rank-sum test to assess whether genes in the curated gene set had significantly lower *P* values (as calculated by the minimum *P* value test) than the matched control genes. When gene sets were combined into ‘obesity’, ‘β-cell function’, ‘other’ and ‘overall’, we selected fivefold matched control genes. Fold enrichment represents the fraction of genes in the gene set that are within the top 10% of associations with youth-onset T2D when compared to background genes.

### LVE analysis

For all calculations of genetic LVE (for a variant or for a gene), we used a previously reported formula that incorporates three genotypes (*AA*, *Aa*, *aa*), their population frequencies and their relative risks (1, RR1 and RR2)^39^. We calculated values for this formula on the basis of observed allele frequency (MAF for single-variant association and cumulative allele frequency for gene-level association) and observed variant or gene effect size (which we converted to relative risk assuming a diabetes prevalence of 8% under an additive genetic model); the extension from variants to genes requires some approximations and assumptions (similar to those of a burden test) and has been previously applied and described in detail^6^.

To compare rare and common variant genetic architectures within ProDiGY, we performed several LVE calculations for both rare variant gene-level associations and common variants. Our primary analysis was to compare LVE explained by ten gene-level associations of tier 3 genes (*MC4R*, *HNF1A*, *GCK*, *SLC30A8*, *ABCC8*, *PAM*, *RFX6*, *GHRL*, *HESX1*, *SIX3*, excluding *ATXN2L* as its association was driven by a common variant) to the ten strongest validated common variant associations of adult-onset T2D (rs2237897, rs7903146, rs150111048, rs1421085, rs4929965, rs11709077, rs10882101, rs1708302, rs738408, rs10937721). As a secondary sensitivity analysis, we conducted additional comparisons using different sets of associations: (1) three gene-level associations and six common variant associations that reached exome-wide significance, (2) the top 25 gene-level associations in ProDiGY and the top 25 common variant associations in ProDiGY and (3) the 38 gene-level associations of tier 4 genes and the top 38 common variant associations in AMP-T2D-GENES.

We also compared the LVE estimates for youth-onset T2D obtained using ProDiGY to those for adult-onset T2D using AMP-T2D-GENES, focusing on rare and common variants. For tier 3 (*n* = 10) and tier 4 (*n* = 38) genes and common variants, the identical genes and variants used in ProDiGY were used to calculate LVE for adult-onset T2D (AMP-T2D-GENES). For exome-wide significant genes and variants, as well as the top 25 genes and variants, the corresponding genes and variants that met the criteria (exome-wide significant or top 25) in adult-onset T2D were used to compute LVE. These exome-wide or top 25 genes and variants underwent winner’s curse correction for both ProDiGY and AMP-T2D-GENES, as detailed below.

These comparisons were subject to two sources of bias and confounding. First, using observed variant or gene effect size estimates upwardly biases LVE calculations, due to uncertainty in effect size estimation. This is particularly problematic when comparing variants of different frequencies (which produce standard errors of different magnitudes) and when comparing variants or genes across studies (for example, ProDiGY and AMP-T2D-GENES) that have substantially different sample sizes. A second source of bias is winner’s curse: many of our analysed variant or gene sets were selected on the basis of achieving a significant association in either ProDiGY or AMP-T2D-GENES. This upwardly biases LVE estimates to a degree that is influenced by both the power of the study and the ascertainment criteria for the set.

We therefore first extended our LVE formula to account for uncertainty in the estimate of effect size for a variant or gene (which upwardly biases LVE estimates). As has been shown previously^40^, for quantitative traits the observed variance explained is approximately equal to the true variance explained plus the square of the standard error in the effect size estimate. We therefore calculated LVE by (1) calculating an upwardly biased LVE estimate using the estimated variant or gene effect size; (2) calculating an LVE correction term using the square of the estimated variant or gene standard error and (3) subtracting the correction term from the upwardly biased estimate. We verified using simulations that this calculation produced unbiased LVE estimates under the null.

For gene-level LVE estimation, we used effect size estimates from the mask achieving the lowest *P* value. We used standard errors corrected for the effective number of masks: we applied our minimum *P* value procedure to convert the original mask *P* value to a corrected *P* value, we converted this *P* value to a *z* score under the normal distribution, and we then computed the standard error that would produce this *z* score given the mask-level effect size.

We finally ensured that we corrected all LVE estimates for winner’s curse by applying our winner’s curse correction (above) to the effect size estimates for variants or genes. This only affected variant or gene sets ascertained on the basis of their observed *P* values or ranks within ProDiGY (or AMP-T2D-GENES); sets ascertained by other criteria were not subjected to winner’s curse correction.

### Common and rare variant scores

To investigate individual-level genetic heterogeneity within ProDiGY, we sought to compare the phenotypes of cases ‘due predominantly to common variants’ to those of cases ‘due predominantly to rare variants’. Ideally, we would identify such individuals by constructing youth-onset T2D common variant and rare variant polygenic scores in an independent cohort, and then applying them to ProDiGY. However, no such independent cohort exists. Therefore, we approximated these by defining rare and common variant ‘contribution scores’ using association statistics within ProDiGY, correcting for winner’s curse in situations when variants were ascertained on the basis of ProDiGY *P* values.

If we were to use these scores to predict risk of youth-onset T2D in ProDiGY, we would be subject to circularity and overfitting due to overlapping training and test data. However, as we only evaluated phenotypes orthogonal to T2D (for example, C-peptide, age of diagnosis, BMI), we reasoned that overfitting would be less of a concern (if any). Nonetheless, we use the term ‘contribution score’ rather than ‘polygenic risk score’ to avoid any implication that we are attempting to apply these in the traditional risk prediction setting.

For the common variant contribution score, we used the same method as previously described for polygenic score^4^. Briefly, we constructed a common variant score for each ancestry using risk alleles and their effect sizes from previously reported T2D GWAS^41^ that did not include samples from ProDiGY and standardized the scores to *z* scores. These *z* scores were converted to *β* values on the basis of the effect size in each ancestry^4^. The *β* values for a one standard deviation increase in common variant score was 0.89, 0.37 and 0.91 for Europeans, African Americans and Latinos, respectively^4^. As these scores were constructed from data independent to ProDiGY, they are unbiased ‘polygenic scores’ for ProDiGY samples rather than simply ‘contribution scores’.

For the rare variant contribution score, we used 46 genes that met two criteria: (1) they were included in at least one of the 25 significantly enriched gene sets and were related to metabolic phenotypes of diabetes, and (2) had at least nominally significant gene-level association with youth-onset T2D (*P* < 0.05). We then applied a previous procedure^42^ for constructing a polygenic score from variants in each mask, applying the ‘unique’ fitting procedure; briefly, each variant is assigned a weight equal to the estimated effect size of the specific mask that contains it, with the effect size computed after removing variants in the mask that were present in more stringent masks. As the genes in this score were selected on the basis of ProDiGY *P* values, we applied a winner’s curse correction (above) to their effect sizes before constructing the score. Combined rare and common variant contribution scores were generated by adding the common variant score and rare variant score for each sample.

We conducted two analyses using these scores. First, we compared the relative number of samples that had common, rare and combined scores above OR ≥ 3. Second, we tested each rare and common score for association with age at diabetes diagnosis, BMI *z* score and log_10_(C-peptide) level in ProDiGY cases using linear regression.

### Ethics statement

All clinical research was approved by the institutional review board of the participating cohort and written informed consent was obtained from each study participant (and their parent or guardian if the participant was under 18 years of age). All clinical investigations were conducted according to the Declaration of Helsinki.

### Reporting summary

Further information on research design is available in the Nature Portfolio Reporting Summary linked to this article.

### Supplementary information

Supplementary Information Complete list of the ProDiGY Consortium working group members.

Reporting Summary

Supplementary Table Supplementary Tables 1–27.

## Data Availability

Sequence data and phenotypes for this study are available via the database of Genotypes and Phenotypes (dbGAP accession IDs phs001533 and phs001511) and the corresponding author upon reasonable request. Most of the raw data are presented in the corresponding Supplementary Tables.

## Code availability

Scripts for (1) calculating LVE, (2) correcting *β* for winner’s curse, Wilcoxon rank-sum test for gene sets and simulating sample overlap are available on github (https://github.com/Soo-Heon/ProDiGY/).
