# Supplementary material for: Genetic architecture and biology of youth-onset type 2 diabetes
Source: Nat Metab. 2024 Jan 26;6(2):226–37. doi: 10.1038/s42255-023-00970-0 (PMC10896722; doi:10.1038/s42255-023-00970-0)
Supplement: Supplementary file 1 — Complete list of the ProDiGY Consortium working group members. [file 42255_2023_970_MOESM1_ESM.pdf]

nature metabolism

Letter https://doi.org/10.1038/s42255-023-00970-0

Genetic architecture and biology of youth-onset type 2 diabetes

In the format provided by the authors and unedited

Complete list of the ProDiGY consortium working group members

ProDiGY

The following individuals constitute the ProDiGY Study Group: M.H. Black (Janssen Pharmaceuticals), B. Burke (George Washington University), L. Chen (Massachusetts General Hospital), D. Dabelea (University of Colorado), J. Divers (New York University), K. Drews (George Washington University), J. Flannick (Boston Children's Hospital & Broad Institute), J.C. Florez (Massachusetts General Hospital & Broad Institute), M. Kelsey (University of Colorado), J. Mercader (Massachusetts General Hospital & Broad Institute), A. Manning (Massachusetts General Hospital & Broad Institute, T. Pollin (University of Maryland), N. Santoro (Yale University), R.Shah (Children's Hospital of Philadelphia), S. Srinivasan (University of San Francisco), J Todd (University of Vermont), P. Zeitler (University of Colorado).

ProDiGY is a collaborative effort to understand the genetic predisposition of youth-onset T2D using multi-ethnic diabetes cases from SEARCH, TODAY, and the TODAY Genetics study. The following individuals and institutions constitute the SEARCH, TODAY and TODAY Genetics Study Groups (* indicates principal investigator or director):

SEARCH for Diabetes in Youth study group

California, USA P Hung, C Koebnick, J M Lawrence*, X Li, E Lustigova (Kaiser Permanente Southern California, Pasadena); D J Pettitt (Santa Barbara)*. North Carolina, USA E J Mayer-Davis*, A Mottl, J Thomas (University of North Carolina, Chapel Hill). South Carolina, USA M Jackson, L Knight, C Turley (University of South Carolina, Columbia); D Bowlby (Medical University of South Carolina, Charleston); J Amrhein, E Apperson, B Nelson (Greenville Health System and Eau Claire Cooperative Health Center, Columbia). Colorado, USA T Crume, D Dabelea*, R F Hamman, A Shapiro, L Testaverde (Colorado School of Public Health, University of Colorado, Denver); G J Klingensmith, D Maahs, M J Rewers, P Wadwa (Barbara Davis Center for Childhood Diabetes, Aurora); S Daniels, M G Kahn, G Wilkening (Children's Hospital Colorado, Aurora); C A Bloch (Pediatric Endocrine Associates, Greenwood Village); J Powell (Indian Health Service, Navajo Area); K Love-Osborne (Denver Health and Hospitals Authority, Denver);

Arizona, USA D C Hu (Tuba City Regional Health Care Center, Tuba City); Ohio, USA L M Dolan*, A Standiford, E M Urbina (University of Cincinnati, Cincinnati); Washington, USA I Hirsch, G Kim, F Malik, L Merjaneh, C Pihoker*, A Roberts, C Taplin, J Yi-Frazier (University of Washington, Seattle); N Beauregard, C Franklin, C Gangan, S Kearns, M Klingsheim, B Loots, M Pascual (Seattle Children's Hospital, Seattle); C Greenbaum (Benaroya Research Institute, Seattle).*D J Pettitt retired in September, 2014.

TODAY and TODAY Genetics study group

Clinical centers Baylor College of Medicine: S. McKay*, M. Haymond*, B. Anderson, C. Bush, S. Gunn, H. Holden, G. Jeha, S.M. Jones, N. Kamith, S. McGirk, N. Miranda, A. Pihlaskari, S. Thamotharan, R. Zagado Case Western Reserve University: L. Cuttler (deceased)*, S. Narasimhan* E. Abrams, T. Casey, W. Dahms (deceased), C. Ievers-Landis, B. Kaminski, M. Koontz, S. MacLeish, P. McGuigan Children's Hospital Central California: S. Banerjee*, A. Bily, N. Delabruere, A. Evaristo Children's Hospital Los Angeles: M. Geffner*, V. Barraza, N. Castaneda, N. Chang, B. Conrad, D. Dreimane, S. Estrada, L. Fisher, E. Fleury-Milfort, S. Hernandez, B. Hollen, F. Kaufman, E. Law, V. Mansilla, D. Miller, C. Muñoz, R. Ortiz, A. Ward, K. Wexler, Y.K. Xu, P. Yasuda Children's Hospital of Philadelphia: L. Levitt Katz*, R. Berkowitz, S. Boyd, N. Chaudhary, C. Clark, K. Gralewski, B. Johnson, P. Kanan, J. Kaplan, C. Keating, C. Lassiter, R. Liillii, T. Lipman, G. McGinley, H. McKnight, B. Schwartzman, R.Shah, R. Volpe, S. Willi Children's Hospital of Pittsburgh: S. Arslanian*, L. Bednarz, F. Bacha, S. Foster, B. Galvin, T. Hannon, A. Kriska, I. Libman, M. Marcus, K. Porter, D. Shearer, T. Songer, E. Venditti Children's Mercy Hospital Kansas City: F. Ugrasbul*, J. Bedard, J. Jacobson, T. Luetjen Columbia University Medical Center: R. Goland*, G. Covington, D. Gallagher, P. Kringas, N. Leibel, D. Ng, M. Ovalles, B. Park, D. Seidman Connecticut Clinical Medical Center Children's Hospital: E. Estrada*, H. Nielsen Emory University: A. Muir*, E. Ivie, P. Jenkins, K. Lindsley Indiana University: Z. Nabhan*, P. Didrick Joslin Diabetes Center: L. Laffel*, A. Benier, A. Goebel-Fabbri, M. Hall, L. Higgins, J. Keady, M. Malloy, K. Milaszewski, L. Rasbach Massachusetts General Hospital: D.M. Nathan*, L. Levitsky*, A. Angelescu, L. Bissett, C. Ciccarelli, L. Delahanty, V. Goldman, O. Hardy, E. Javier, M. Larkin, R. McEachern, D. Norman, D. Nwosu, S. Park-Bennett, R. Pompei, D. Richards, N. Sherry, B. Steiner Saint Louis University: S. Tollefsen*, S. Carnes, D. Dempsher, D. Flomo, T. Whelan, B. Wolff State University of New York Upstate Medical University: R. Weinstock*, R. Izquierdo, D. Bowerman, S. Bzdick, J. Bulger, P. Conboy, J. Hartsig, R. Izquierdo, J. Kearns, R. Saletsky, P. Trief Texas Tech University: D. Dreimane*, C. Lovett, C. Scott-Johnson

University of Arkansas: A. Morales*, C. Moreau University of California San Diego: M. Gottschalk* University of Colorado Denver: P. Zeitler* (Steering Committee Chair), N. Abramson, A. Bradhurst, N. Celona-Jacobs, M. Downey, J. Higgins, A. Hull, M.M. Kelsey, G. Klingensmith, R. Morehead, K. Nadeau, H. Tyrrell, T. Witten University of Florida: J. Silverstein*, E. Dougherty, T. Lyles University of Oklahoma Health Sciences Center: K. Copeland* (Steering Committee Vice-Chair), E. Boss, R. Brown, J. Chadwick, L. Chalmers, S. Chernausek, A. Hebensperger, C. Macha, R. Newgent, A. Nordyke, D. Olson, T. Poulsen, L. Pratt, J. Preske, J. Schanuel, S. Sternlof, M. Zarate University of Texas Health Science Center at San Antonio: J. Lynch*, D. Hale*, N. Amodei, R. Barajas, C. Cody, J. Hernandez, C. Ibarra, E. Morales, S. Rivera, G. Rupert, A. Wauters Vanderbilt University: A. Potter*, M. Black, F. Brendle, A. Shannon Washington University in St Louis: N. White*, A. Arbeláez, D. Flomo, J. Jones, T. Jones, M. Sadler, A. Starnes, M. Tanner, A. Timpson, R. Welch Yale University: S. Caprio*, E. Duran, M. Grey, C. Guandalini, S. Lavietes, P. Rose, A. Syme, W. Tamborlane

Coordinating center George Washington University Biostatistics Center: K. Hirst*, B. Burke, S. Edelstein, P. Feit, N. Grover, C. Long, L. Pyle

Project office National Institute of Diabetes and Digestive and Kidney Diseases: B. Linder*

Central units Central Blood Laboratory (Northwest Lipid Research Laboratories, University of Washington): S.M. Marcovina*, J. Harting, M. Ramirez, G. Strylewicz University of Maryland School of Medicine: T. Pollin*, A Shuldiner*
