# Supplementary material for: Genetic architecture and biology of youth-onset type 2 diabetes
Source: Nat Metab. 2024 Jan 26;6(2):226–37. doi: 10.1038/s42255-023-00970-0 (PMC10896722; doi:10.1038/s42255-023-00970-0)
Supplement: Supplementary file 2 — Reporting Summary [file 42255_2023_970_MOESM2_ESM.pdf]

nature portfolio

Corresponding author(s): Jason Flannick

Last updated by author(s): Dec 14, 2023

Reporting Summary

Nature Portfolio wishes to improve the reproducibility of the work that we publish. This form provides structure for consistency and transparency in reporting. For further information on Nature Portfolio policies, see our Editorial Policies and the Editorial Policy Checklist.

Statistics

For all statistical analyses, confirm that the following items are present in the figure legend, table legend, main text, or Methods section.

n/a	Confirmed	
☐	☒	The exact sample size (n) for each experimental group/condition, given as a discrete number and unit of measurement
☐	☒	A statement on whether measurements were taken from distinct samples or whether the same sample was measured repeatedly
☐	☒	The statistical test(s) used AND whether they are one- or two-sided<br>*Only common tests should be described solely by name; describe more complex techniques in the Methods section.*
☐	☒	A description of all covariates tested
☐	☒	A description of any assumptions or corrections, such as tests of normality and adjustment for multiple comparisons
☐	☒	A full description of the statistical parameters including central tendency (e.g. means) or other basic estimates (e.g. regression coefficient) AND variation (e.g. standard deviation) or associated estimates of uncertainty (e.g. confidence intervals)
☐	☒	For null hypothesis testing, the test statistic (e.g. F, t, r) with confidence intervals, effect sizes, degrees of freedom and P value noted<br>*Give P values as exact values whenever suitable.*
☒	☐	For Bayesian analysis, information on the choice of priors and Markov chain Monte Carlo settings
☒	☐	For hierarchical and complex designs, identification of the appropriate level for tests and full reporting of outcomes
☐	☒	Estimates of effect sizes (e.g. Cohen's d, Pearson's r), indicating how they were calculated

Our web collection on statistics for biologists contains articles on many of the points above.

Software and code

Policy information about availability of computer code

Data collection	N/A
Data analysis	Scripts for 1) calculating liability variance explained, 2) correcting beta for winner's curse, Wilcoxon rank sum test for gene sets, and simulating sample overlap are available on github (https://github.com/Soo-Heon/ProDiGY/).

For manuscripts utilizing custom algorithms or software that are central to the research but not yet described in published literature, software must be made available to editors and reviewers. We strongly encourage code deposition in a community repository (e.g. GitHub). See the Nature Portfolio guidelines for submitting code & software for further information.

Data

Policy information about availability of data

All manuscripts must include a data availability statement. This statement should provide the following information, where applicable:

- Accession codes, unique identifiers, or web links for publicly available datasets
- A description of any restrictions on data availability
- For clinical datasets or third party data, please ensure that the statement adheres to our policy

Sequence data and phenotypes for this study are available via the database of Genotypes and Phenotypes (dbGAP accession ID: phs001533 and phs001511) and the corresponding author upon reasonable request.

Research involving human participants, their data, or biological material

Policy information about studies with human participants or human data. See also policy information about sex, gender (identity/presentation), and sexual orientation and race, ethnicity and racism.

Reporting on sex and gender	Sex-based analysis was not performed and reported, as previous studies have suggested that sex-specific genetic associations do not play a significant role in the development of type 2 diabetes.
Reporting on race, ethnicity, or other socially relevant groupings	Ancestry was determined using genetic principal components, and genetic association analysis for youth-onset type 2 diabetes was conducted within each specific ancestry subgroup. Ancestry was classified into three categories: African American, European, and Hispanic.
Population characteristics	Participants with youth-onset (age < 20) type 2 diabetes was recruited.
Recruitment	ProDiGY is a collaborative effort to understand the genetic predisposition of youth-onset T2D using multi-ethnic diabetes cases from SEARCH, TODAY, and the TODAY Genetics study as previously described1,2. In brief, SEARCH is a longitudinal observation study on youth-onset diabetes in U.S. (diagnosed at < 20 years of age) initiated in 2000. The TODAY study is a randomized clinical trial that enrolled T2D cases with age 10-17 years between 2004-2009. Participants were diagnosed with T2D before 18 years of age; had BMI >= 85th percentile for age, sex, and height; and did not have evidence of type 1 diabetes (negative of pancreatic islet auto-antibodies and positive for C-peptide level > 0.6 ng/mL). The TODAY Genetics study is ancillary to the TODAY clinical trial and enrolled additional cases with similar criteria as the TODAY study.
Ethics oversight	All clinical research was approved by the institutional review board of the participating cohort and written informed consent was obtained from each study participant (and their parent or guardian if the participant was under 18 years of age). All clinical investigations were conducted according to the Declaration of Helsinki.

Note that full information on the approval of the study protocol must also be provided in the manuscript.

Field-specific reporting

Please select the one below that is the best fit for your research. If you are not sure, read the appropriate sections before making your selection.

☒ Life sciences ☐ Behavioural & social sciences ☐ Ecological, evolutionary & environmental sciences

For a reference copy of the document with all sections, see nature.com/documents/nr-reporting-summary-flat.pdf

Life sciences study design

All studies must disclose on these points even when the disclosure is negative.

Sample size	After matching cases and controls based on their genetic background, there were total of 3,005 youth-onset T2D cases and 9,777 controls available for genetic association testing. The effective sample size of this study, defined as 4 × Ncases × Ncontrols / (Ncases + Ncontrols), was 9,194.
Data exclusions	For the analyses, all case and control samples that were carefully matched for genetic background were included.
Replication	Due to the uniqueness of our youth-onset T2D cases, there were no other cohorts with large-scale exome sequence data available for replication purposes. Nevertheless, we attempted to compare our findings with those observed in adult-onset T2D to gain additional insights and contextualize our results.
Randomization	This was a case-control genetic association study that is not subjected for randomization
Blinding	This was a case-control genetic association study and blinding is not applicable.

Reporting for specific materials, systems and methods

We require information from authors about some types of materials, experimental systems and methods used in many studies. Here, indicate whether each material, system or method listed is relevant to your study. If you are not sure if a list item applies to your research, read the appropriate section before selecting a response.

Materials & experimental systems

n/a	Involved in the study
☒	☐ Antibodies
☒	☐ Eukaryotic cell lines
☒	☐ Palaeontology and archaeology
☒	☐ Animals and other organisms
☒	☐ Clinical data
☒	☐ Dual use research of concern
☒	☐ Plants

Methods

n/a	Involved in the study
☒	☐ ChIP-seq
☒	☐ Flow cytometry
☒	☐ MRI-based neuroimaging
